# Direct and indirect effects of age on interoceptive accuracy and awareness across the adult lifespan

**DOI:** 10.3758/s13423-017-1339-z

**Published:** 2017-07-06

**Authors:** Jennifer Murphy, Hayley Geary, Edward Millgate, Caroline Catmur, Geoffrey Bird

**Affiliations:** 1 Social, Genetic and Developmental Psychiatry Centre (MRC), Institute of Psychiatry, Psychology and Neuroscience - PO80, King’s College London, De Crespigny Park, Denmark Hill, London, SE5 8AF UK; 2 Institute of Psychiatry Psychology and Neuroscience, Department of Psychology, King’s College London, PO Box 78, 4 Windsor Walk, London, SE5 8AF UK; 3 Department of Experimental Psychology, University of Oxford, Oxford, UK

**Keywords:** Ageing, Interoceptive accuracy, Interoceptive awareness

## Abstract

Various aspects of physical and mental health have been linked to an individual’s ability to perceive the physical condition of their body (‘interoception’). In addition, numerous studies have demonstrated a role for interoception in higher-order cognitive abilities such as decision-making and emotion processing. The importance of interoception for health and typical cognitive functioning has prompted interest in how interoception varies over the lifespan. However, few studies have investigated interoception into older adulthood, and no studies account for the set of physiological changes that may influence task performance. The present study examined interoception from young to very late adulthood (until 90 years of age) utilising a self-report measure of interoception (Study One) and an objective measure of cardiac interoception (Study Two). Across both studies, interoception decreased with age, and changes in interoceptive accuracy were observed which were not explained by accompanying physiological changes. In addition to a direct effect of age on interoception, an indirect effect of ageing on cardiac interoceptive accuracy mediated by body mass index (BMI) was found, such that ageing was associated with increased BMI which was, in turn, associated with reduced interoceptive accuracy. Such findings support and extend previous research demonstrating interoceptive decline with advancing age, and highlight the importance of assessing whether decreasing interoceptive ability is responsible for some aspects of age-related ill-health and cognitive impairment.

## Introduction

Interoception is described as the perception of the physical condition of the body (Craig, 2002), including numerous visceral sensations such as hunger, itch, respiratory and cardiac awareness (Khalsa & Lapidus, 2016). Whilst accurate perception of bodily sensations may be crucial for homeostasis, and therefore for maintaining physical health, in recent years there has been a growing interest in the relationship between interoception and higher-order cognition. Indeed, interoception has been related to various aspects of cognition, including emotional processing (Füstös, Gramann, Herbert, & Pollatos, 2013; Schandry, 1981; Terasawa, Fukushima, & Umeda, 2013; Wiens, Mezzacappa, & Katkin, 2000) and risky decision making (Dunn et al., 2010a; Sokol-Hessner, Hartley, Hamilton, & Phelps, 2015; Werner, Jung, Duschek, & Schandry, 2009), supporting theories of emotion and decision making that ascribe a fundamental role for the perception of bodily sensations (Critchley & Nagai, 2012; Damasio, 1994; Garfinkel & Critchley, 2013; Gendron & Barrett, 2009; Schachter & Singer, 1962; Seth, 2013). Furthermore, evidence supporting a role for interoception in higher-order cognition is consistent with both empirical evidence (e.g., (Ardizzi et al., 2016; Ehlers & Breuer, 1992; Gaigg, Cornell & Bird, 2016; Garfinkel, et al., 2016a; Herbert, Herbert, & Pollatos, 2011; Herbert & Pollatos, 2014; Klabunde, Acheson, Boutelle, Matthews, & Kaye, 2013; Mussgay, Klinkenberg, & Rüddel, 1999; Pollatos et al., 2008; Shah, Hall, Catmur, & Bird, 2016) and theoretical models (Brewer, Happé, Cook, & Bird, 2015; Ehlers, 1993; Harshaw, 2015; Khalsa & Lapidus, 2016; Murphy, Brewer, Catmur, & Bird, 2017; Naqvi & Bechara, 2010; Paulus & Stein, 2006; Quattrocki & Friston, 2014; Verdejo-Garcia, Clark, & Dunn, 2012) linking aberrant interoception (both atypically high and low interoception; see Murphy et al., 2017) to poor mental health in conditions such as panic and anxiety disorders, feeding and eating disorders, substance abuse, autism, schizophrenia, somatic symptom disorders and alexithymia.

Given this body of evidence linking interoception to aspects of higher-order cognition and physical and mental health (Brewer, Cook, & Bird, 2016; Khalsa & Lapidus, 2016; Murphy et al., 2017), examining the stability of interoception across an individual’s lifespan may be crucial. Notably, it has been found that cardiac interoceptive accuracy declines with age (Khalsa, Rudrauf, & Tranel, 2009), supporting earlier work reporting that the perception of other interoceptive signals, including thirst (see Silver, 1990), taste (Stevens, Cruz, Hoffman, & Patterson, 1995), temperature (Clark & Mehl, 1971) and pain (see Gagliese, 2009), is less accurate in later life (but see Garfinkel et al., 2016b). However, although a limited number of studies have therefore looked at developmental influences on the *accuracy* of interoception, to our knowledge no study has examined age-related changes in self-reported *awareness* of interoceptive signals (known as interoceptive sensibility under the framework of Garfinkel, Seth, Barrett, Suzuki, & Critchley, 2015), which is of interest as self-report measures of interoceptive awareness do not always correlate with objective tests of interoceptive accuracy (Garfinkel et al., 2015). Study One therefore aimed to examine interoceptive awareness across the lifespan.

Alongside the investigation of whether interoceptive awareness declines over the lifespan, there is a need to improve our understanding of changes in interoceptive accuracy. Studies examining changes in interoceptive accuracy across the lifespan typically use one of the two most commonly used measures of interoception: heartbeat tracking (Schandry, 1981) and discrimination procedures (Brener & Kluvitse, 1988; Whitehead, Drescher, Heiman, & Blackwell, 1977), which both assess perception of cardiac signals. In the former, participants are asked to count their heartbeats over a series of intervals whilst their heartbeat is objectively measured. The difference between the objective and subjective measurements acts as a measure of interoceptive accuracy. In the latter, participants are asked to determine whether a signal is in or out of sync with their heartbeat. In a study by Khalsa and colleagues (2009), age explained ~40% of the variance in heartbeat discrimination performance in participants aged up to 63 years of age, such that increasing age was associated with poorer performance.

Examining cardiac interoception across the lifespan is complicated, however, by previous findings demonstrating that various physiological and psychological factors influence task performance, and have also been associated with ageing. For example, lower resting heartrate, reduced heartrate variability and body composition (e.g., lower BMI/body fat) have all been associated with better cardiac perception (Knapp-Kline & Kline, 2005; Rouse, Jones, & Jones, 1988), while reduced systolic blood pressure has been linked to worse cardiac perception (O’Brien, Reid, & Jones, 1998). Psychological factors such as participants’ beliefs regarding heartrate have also been shown to strongly influence performance on objective tests of cardiac interoceptive accuracy (Ring, Brener, Knapp, & Mailloux, 2015; Ring & Brener, 1996; Windmann, Schonecke, Fröhlig, & Maldener, 1999) and time perception has also been linked with task performance (see Wittmann, 2013).

It is therefore unclear whether ageing is associated with changes in interoceptive accuracy independent of its effects on these psychological and physiological factors, or whether in fact these factors mediate the effect of age on cardiac interoceptive accuracy (Franklin et al., 1997; St-Onge, 2005; Turgeon, Lustig, & Meck, 2016; Umetani, Singer, McCraty, & Atkinson, 1998; Yashin et al., 2006). Examining whether changes in interoception across the lifespan are attributable directly to aging, or to factors co-varying with age, may be crucial for identifying the mechanism underlying interoceptive change with increased age. Uncovering this mechanism is of interest given the relationship between interoception and socio-cognitive abilities (Murphy et al., 2017), and the well-documented decline in these abilities in later life (e.g., Ruffman, Henry, Livingstone, & Phillips, 2008; Sparrow & Spaniol, 2016). Furthermore, although a decline in heartbeat discrimination performance with age has been demonstrated in participants aged up to 63 years of age (Khalsa et al., 2009), whether cardiac interoceptive accuracy continues to decline after this period into late, and very late, adulthood remains an unanswered question, and is the focus of Study Two.

The present pair of studies therefore aimed to quantify interoceptive awareness (Study One) and accuracy (Study Two) across the lifespan, from young adulthood to very late adulthood. Study One utilised self-report measures, whereas Study Two utilised objective measures whilst also measuring psychological and physiological factors which may mediate any association between cardiac interoception and age.

## Study one

### Methods

#### Participants

A total of 1008 participants took part in an online survey with a prize draw offered as incentive. Of these, 898 participants completed the survey as part of a larger study that included additional questionnaires, with the remainder (110 participants) only completing the interoceptive awareness questionnaire. Participants were recruited from pre-existing participant databases and via social media outlets. Of the 1008 participants, 345 participants fully completed the survey, reported no pre-existing psychiatric conditions and had English as their first language and were therefore included in the study (*M*_*age*_ = 38.66, *SD*_*age*_ = 17.59, Age range 18–89 years, 95 males, 0 other). Of the 345 participants, 235 completed the additional questionnaires. In line with the declaration of Helsinki, all participants gave informed consent and were debriefed upon task completion.

#### Measures and procedure

The online survey was conducted using Qualtrics Research Suite (Qualtrics, Provo, UT, USA). To quantify interoceptive awareness, the very short version of the Body Perception Questionnaire (BPQ; Porges, 1993; Kolacz et al., in preparation) was used. In this 12-item questionnaire, participants are asked to indicate on a 5-point Likert scale (strongly disagree to strongly agree) whether they are aware of particular bodily sensations (e.g., their mouth being dry) during most situations. Prior to the questionnaire, demographic details were collected, including age (years) and sex (male, female, other). Additionally, participants were asked to indicate whether English was their first language and whether they had a diagnosis of any psychiatric conditions.

#### Results and discussion

**Fig. 1:**
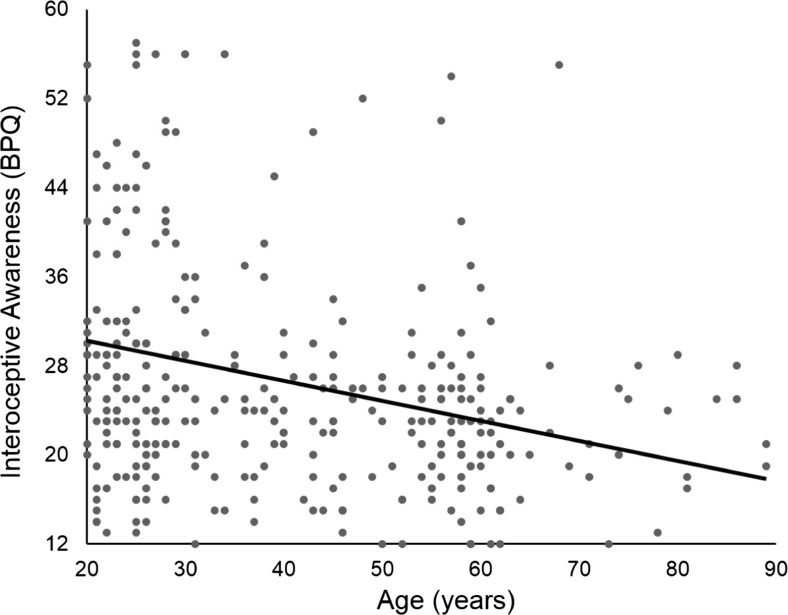
A significant relationship between self-reported interoceptive awareness, as measured by the very short BPQ, and age was observed such that increasing age was associated with poorer interoceptive awareness

BPQ scores ranged from 12 to 57 (*M* = 26.89, *SD* = 10.12) with high scores representing greater self-reported awareness of interoceptive signals. A Spearman’s rank order correlation indicated a significant negative correlation between age (years) and BPQ scores, *r*(345) = –.337, *p* < .001 (two-tailed; Fig. 1). No sex differences were found, *t*(343) = .208, *p* > .250, *d* = .03, 95% CI for *d* (–0.211, 0.261). No differences in BPQ scores were observed between participants completing just the interoception questionnaire and those completing additional questionnaires when age was statistically controlled for, *t*(343) = 0.68, *p* > .250, *d* = .08, 95% CI for *d* (–.154, .317).

A significant negative association between age and self-reported interoceptive awareness was found. These results suggest that interoceptive awareness deteriorates across the lifespan and continues to decline into late, and very late, adulthood. However, given previous findings of a dissociation between subjective tests of interoceptive awareness and objective tests of interoceptive accuracy (Garfinkel et al., 2015), it remains unclear whether the continuing decline of interoceptive awareness into very late adulthood would be accompanied by decreasing interoceptive accuracy as measured by objective tests. To examine this question, Study Two utilised an objective measurement of interoceptive accuracy to quantify interoception across the lifespan.

## Study two

### Methods

#### Participants

A total of 140 participants took part in this study in exchange for a small honorarium. Participants were selected on the basis that they had no known psychiatric or neurological conditions. To screen for cognitive impairment, all participants were administered the Mini Mental State Examination test (MMSE; Folstein, Folstein, & McHugh, 1975), with scores below 23 indicative of cognitive impairment (Tombaugh & McIntyre, 1992). Four participants were excluded (one scored below the typical threshold on the MMSE; two disclosed existing psychiatric/neurological conditions post-testing; and one was excluded due to equipment failure) resulting in 136 valid cases (*M*_*age*_ = 55.10, *SD*_*age*_ = 19.50; age range 20–90 years, 49 males). A minimum of 5 participants were present in each 5-year age bracket. In line with the declaration of Helsinki, all participants gave informed consent and were debriefed upon task completion. To minimise the effects of elevated heartrate on accuracy (Knapp-Kline & Kline, 2005), all participants were asked to refrain from caffeine for 6 h prior to testing.

#### Interoception

The heartbeat tracking task (Schandry, 1981) was used to quantify interoceptive accuracy with timing ability employed as a control task (Ainley, Brass, & Tsakiris, 2014; Shah et al., 2016). Objective heartbeat was measured using a pulse oximeter (Contec Systems, CMS-50Dþ; Qinhuangdao, China) attached to the participant’s right index finger. Each participant completed both the heartbeat tracking task and the timing task over four durations. Two sets of durations were used (either 25, 35, 45, 100 s or 28, 38, 48, 103 s) and the duration sets were counterbalanced across participants such that half of the participants completed the longer durations for the timing task and half completed the longer intervals for the heartbeat task. Additionally, task order was fully counterbalanced, and the order of individual durations was counterbalanced according to a Latin-square across participants. Task order did not affect performance in interoceptive accuracy, *t*(134) = 1.640, *p* > .05, *d* = 0.28, 95% CI for *d* (–0.057, 0.619) or timing accuracy, *t*(134) = 1.364, *p* > .05, *d* = 0.23, 95% CI for *d* (-0.104, 0.571).

During the task, participants were seated with both feet flat on the floor and both hands on the table. Participants were instructed that they would be asked to silently count their heartbeats over a period without physically measuring their heartbeat. With their eyes closed, they were asked to count their heartbeats from when the experimenter said “start” until they heard a beep, at which point they should indicate the number they had counted. They were explicitly told to only count heartbeats they felt and not to count seconds or guess. They were also told that if they did not feel anything they should give zero as their answer. Participants were then given 2 min to practice prior to the first heartrate trial; no feedback was provided. The timing task was identical to the heartbeat task except participants were asked to count seconds rather than their heartbeats.

#### Anthropometrics

For each participant body mass index (BMI) and blood pressure measurements were taken. Blood pressure was taken using an electronic upper arm monitor (Omron M2) whilst participants were seated.

#### Beliefs regarding heartrate

To quantify beliefs, participants were asked to estimate the average person’s resting heartbeat. Specifically, participants were asked ‘How many times do you think the average person’s heartbeats, in 1 min when they are at rest?’ Note that earlier studies sometimes required the participant to estimate their own heartrate (Ring, et al., 2015; Ring & Brener, 1996); this was avoided in the present study to avoid any effect of the estimation on the heartbeat tracking task or vice versa.

### Scoring and data analysis

#### Interoceptive and timing accuracy

Interoceptive accuracy on the heartbeat tracking task was estimated on a scale from 0 to 400: Σ[1 – (|Actual number of heartbeats – participant’s estimate|/Actual number of heartbeats)] × 100. Higher scores indicate better performance (Shah et ﻿al., 2016; see als﻿﻿o Garfinkel et al., 2015). Timing scores were estimated similarly, Σ[1 – (|Actual number of seconds – participant’s estimate|/Actual number of seconds)] × 100. Again, high scores indicate better performance. Average ratio scores (participants’ estimate/objective measure) were also computed with scores above one indicative of overestimation. Unsurprisingly, given the explicit task instructions (see Methods), >95% of participants underestimated the number of heartbeats and a similar pattern was observed for timing accuracy (>80%). This variable was therefore not considered further.

#### Body composition

BMI was calculated using the following equation: mass(kg)/(height(m))^2^.

#### Beliefs regarding heartrate

The accuracy of participants’ beliefs was calculated as a continuous variable calculated by taking the absolute difference between participants’ estimates and the grand mean of resting heartrate reported in large studies of human physiology (Agelink et al., 2001; Ramaekers, Ector, Aubert, Rubens, & Van de Werf, 1998; grand mean = 72.26 beats per minute; bpm). As this variable indicates how far participants’ estimates are from the grand mean, higher scores indicate more inaccurate beliefs about the average person’s heartrate.

#### Heartrate physiology

A proxy of heartrate variability was calculated from pulse-rate signals. This method has been shown to be reliable when participants are at rest (Schäfer & Vagedes, 2013). For each participant, the root mean squared of successive differences (RMSSD) was calculated from the last 60 s of heartrate recording for the longest interval examined (100 or 103 s). RMSSD was favoured over other measures of heartrate variability due to evidence attesting to its better reliability over short durations (Munoz et al., 2015). From this same 60-s interval, mean resting heartrate (average bpm) was taken. For three participants, the recording for the 100-s interval was corrupted, and a 60-s recording from one of the other intervals was used as a replacement which was comparable to the 100-s recordings.

#### Analysis strategy

Zero-order correlations revealed the associations between ageing and cardiac interoceptive accuracy, and ageing and the timing control task. The size of these correlations were compared using Steiger’s *Z*-test (Steiger, 1980) using the quantpsy web implementation (Lee & Preacher, 2013). Correlations were then used to examine the association between age and the possible psychological and physiological mediators of the effect of age on interoceptive accuracy, before similar analyses compared the relationship between these potential mediators and interoceptive accuracy.

The existence of direct and indirect effects of age on interoceptive accuracy was investigated using a parallel mediation model in which BMI, gender, systolic blood pressure, accuracy of beliefs about average heartrate, heartrate variability, mean heartrate, and timing accuracy were entered as potential mediators of the effect of age on interoception. Mediation modelling was carried out using the SPSS macro-script (Process) provided by Hayes (2013) and Preacher and Hayes (2008). For indirect effects, 90% (one-tailed) bias-corrected bootstrapped confidence intervals were calculated using 5,000 repetitions. This method was selected over the Sobel (1982) method as the former does not require the assumption of a normal distribution and simulation studies indicate higher power whilst controlling for Type one error rates (MacKinnon, Lockwood, Hoffman, West, & Sheets, 2002; Mackinnon, Lockwood, & Williams, 2004). As outlined by Preacher and Hayes (2004), an indirect effect is significant if the confidence intervals for the indirect effect do not include zero. For all analyses where directional hypotheses are made, one-tailed *p* values are reported. Standardized coefficients are reported in the mediation analysis.

#### Results and discussion

A small amount of randomly-distributed data were missing (0.74%; three blood pressure measurements, two belief estimates, and one measure of heartrate variability and resting heartrate) and these missing values were imputed using multiple imputation in SPSS. The Mersenne–Twister algorithm with a starting point fixed to 2,000,000 was utilised for random number generation. All variables were entered into the model, the automatic method was selected, and all variables were used as predictors. No participant had more than one missing data point.

Simple zero-order correlations revealed a significant negative association between interoceptive accuracy and age, *r*(136) = –.21 *p* = .008 (one-tailed). Correlations between interoceptive accuracy and timing performance, *r*(136) = .12, *p* > .05 (one-tailed) were not significant, but the correlation between age and timing performance approached significance *r*(136) = –.14, *p* = .05. When formally compared, the sizes of the correlations between ageing and interoceptive accuracy, and ageing and the timing control task, were not significantly different (*z* = 0.6, *p* = .55).

Age was positively associated with BMI, *r*(136) = .25, *p* = .002 (one-tailed). A negative relationship between age and heartrate variability was also observed, *r*(136) = –.16, *p* = .036 (one-tailed). In contrast, age was not associated with resting heartrate, *r*(136) = –.01, *p* > .05 (one-tailed), systolic blood pressure, *r*(136) = –.10, *p* > .05 (one-tailed), or beliefs (*r*(136) = –.04, *p* > .05; two-tailed).

Negative associations were observed between interoceptive accuracy and BMI, *r*(136) = –.19, *p* = .015, and beliefs, *r*(136) = –.232, *p* = .003 (one-tailed). A positive association was observed between interoceptive accuracy and systolic blood pressure, *r*(136) = .21, *p* = .008 (one-tailed). No association was observed between interoception and heartrate variability, *r*(136) = .06, *p* > .05, or resting heartrate, *r*(136) = –.10, *p* > .05 (one-tailed).

**Fig. 2:**
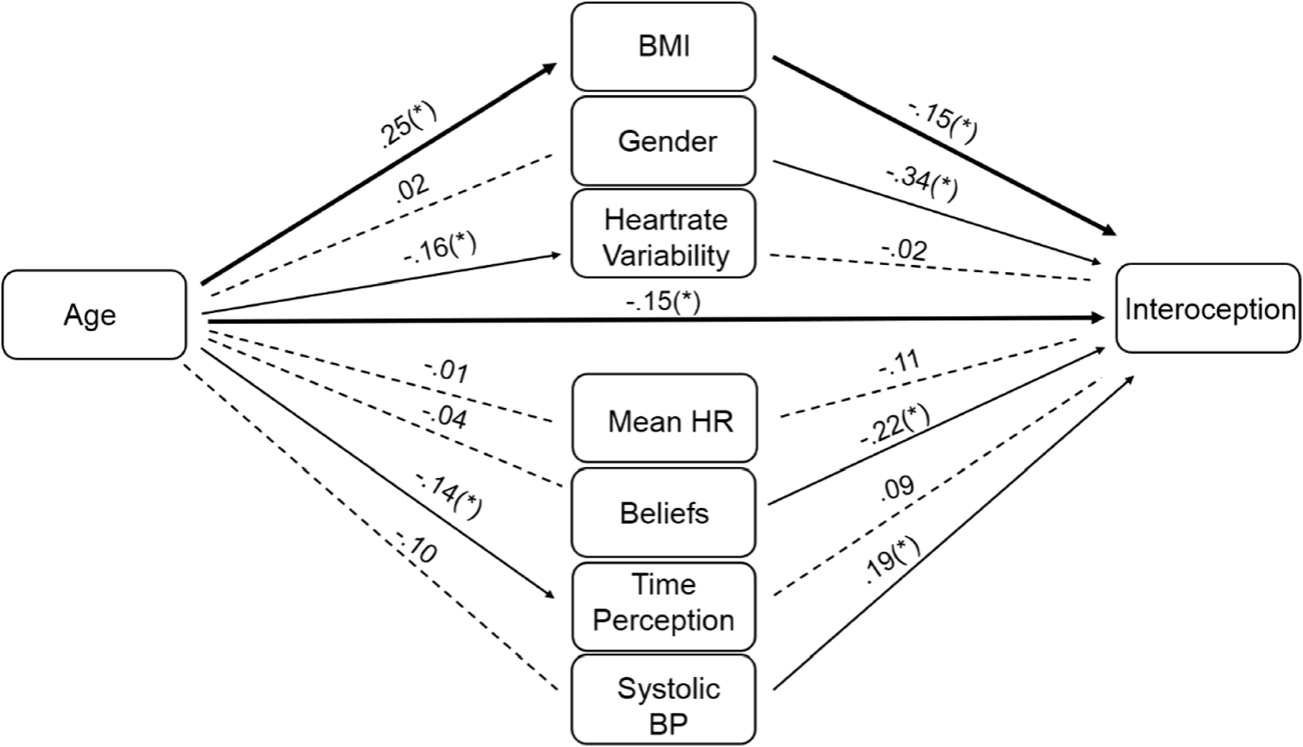
Depicts the results of the mediation analysis. Mediation analysis indicated a significant path from age to BMI, heartrate variability and time perception ability (a path; the individual relationships between the IV and the mediators﻿; e.g., from age to BMI; Fig. 2 left), whilst significant paths to interoceptive accuracy from BMI, gender, heartrate variability beliefs and time perception ability were also observed (b path; the relationship between the mediators and the DV controlling for other mediators and keeping the IV constant; e﻿.g., from BMI to interoceptive accuracy; Fig. 2 right). Most importantly, a direct effect of age on interoceptive accuracy was observed (c’ path; the relationship between the IV and the DV controlling for the mediators; from age to interoceptive accuracy; Fig. 2 centre), in addition to an indirect path from age to interoception via BMI. The indirect path via BMI thus partially mediated the effect of age on interoception. *Denotes significance at the *p* < .05 level (one-tailed). *BMI* Body Mass Index; *Gender* Male or Female (0 or 1); *Heartrate Variability* variation in the time interval between heartbeats; *Mean HR* The average second-by-second heartbeat recorded over 60 seconds; *Beliefs* the error of participants’ estimates regarding the average heartrate; *Time Perception* Performance on the time estimation task; *Systolic Blood Pressure* Taken whilst seated and measured in millimetres of mercury

The results of the mediation model are illustrated in Fig. 2. As can be seen, there was a significant effect of age on BMI, heartrate variability, and performance on the timing control task (as outlined above; all one-tailed). All other predictors were non-significant (all *p* > .10). There were also significant effects of BMI (*b* = –.15, *SE* = .09, *p* = .04), gender (*b* = –.34, *SE* = .17, *p* = .02), beliefs regarding average heartrate (*b* = –.22, *SE* = .08, *p* = .004) and systolic blood pressure (*b* = .19, *SE* = .08, *p* = .01) on interoception (all one-tailed). All other predictors were non-significant (all *p* > .10). Of importance was the finding that age exerted a direct (i.e. unmediated) effect on interoception (direct effect = –.15, *SE* = .09, *t* = 1.69, *p* = .047; one-tailed) and a small indirect effect on interoception mediated by the effect of BMI (*b* = –.039, *SE* = .03, CI (–.982, –.004); one-tailed) despite no total indirect effect (*b* = –.06, *SE* = .05, CI (–1.12, .25). All other indirect effects were non-significant (e.g., confidence intervals included zero). The indirect effect of BMI accounted for 0.18 (18%) of the total effect between age and interoception (*ab/c*; see Jose, 2013), indicating partial mediation.

## General discussion

This set of studies aimed to examine variation in interoceptive awareness and accuracy across the lifespan. In Study One, a negative relationship between age and interoceptive awareness was observed; older participants rated their interoceptive awareness as reduced. Building on this, Study Two examined cardiac interoceptive accuracy across the adult lifespan using an objective performance measure, demonstrating a negative relationship between age and interoceptive accuracy. These data confirm previously reported evidence suggesting a decline in interoceptive accuracy in older adulthood (Khalsa et al., 2009), and go further to suggest that this decline continues into both late and very late adulthood.

The results from Study Two highlight that the poor performance observed in late adulthood is due to both a direct effect of age on interoceptive accuracy and an indirect effect mediated by BMI, whereby age was associated with increased BMI, which in turn, predicted reduced interoceptive accuracy. Therefore, it appears that changes in BMI partially mediate the relationship between age and interoception. Other physiological and psychological changes such as heartrate variability, resting heartrate, and time perception, previously associated both with interoceptive accuracy (Knapp-Kline & Kline, 2005; O’Brien et al., 1998; Rouse et al., 1988; Wittmann, 2013) and increasing age (Franklin et al., 1997; St-Onge, 2005; Umetani et al., 1998; Yashin et al., 2006), were not significant mediators of the age–interoception relationship.

The partial mediation of the effect of age on interoceptive accuracy by BMI provides some indication as to the mechanism by which ageing negatively impacts interoceptive accuracy. However, the existence of a direct effect of age indicates at least one further mechanism to be identified. Although the results of Study One demonstrated reduced awareness of interoceptive information with increasing age, previous reports of a dissociation between self-reported interoceptive awareness and interoceptive accuracy (Garfinkel et al., 2015) mean that care must be taken in interpreting reduced awareness of interceptive signals as underlying the direct effect of age on interoceptive accuracy.

The relationship between age and performance on the interoceptive accuracy and timing control tasks is worthy of note. Whereas the zero-order correlations indicated a similar magnitude of the effect of ageing on performance of these tasks, the results of the mediation analysis demonstrate that the effects of ageing on interoceptive accuracy were not a product of reduced timing/counting ability (Turgeon et al., 2016; Wittmann, 2013), and are also likely not due to general problems with motivation or attention. Thus, although similar age-related declines were observed across these tasks, the effects of age were independent.

The finding that interoception declines throughout the lifespan raises important questions regarding the extent to which interoception is related to the decline in socio-emotional competence and altered cognition observed in late adulthood (Murphy et al., 2017). For example, a body of research indicates poorer emotion recognition (Ruffman et al., 2008) and changes in risky decision making with advancing age (Sparrow & Spaniol, 2016). Crucially, these same abilities have been linked to interoceptive accuracy (Dunn et al., 2010b; Füstös et al., 2013; Schandry, 1981; Sokol-Hessner et al., 2015; Terasawa et al., 2013; Werner et al., 2009; Wiens et al., 2000). Given the interrelatedness of these factors, determining the extent to which age-related changes in these abilities is predicted by interoception is an important aim for future research. Moreover, if interoception does underlie adverse age-related cognitive change, then such evidence may inform interventions, such as interoceptive training (e.g., Canales-Johnson et al., 2015; Schaefer, Egloff, Gerlach, & Witthöft, 2014; Schandry & Weitkunat, 1990) designed to ameliorate undesirable effects of ageing on cognition and socio-emotional competence.

In comparison to the only other study examining the impact of older age on cardiac interoceptive accuracy (Khalsa et al., 2009), the variance explained by age in the present study was modest. The difference in the size of the observed age effect could be because the present study quantified interoceptive accuracy using the heartbeat tracking task (Schandry, 1981), whereas Khalsa et al. (2009) utilised the heartbeat discrimination procedure (Brener & Kluvitse, 1988; Whitehead et al., 1977). Whilst small to moderate correlations have been observed between these two tasks (Garfinkel et al., 2015; Knoll & Hodapp, 1992; but see Phillips, Jones, Rieger, & Snell, 1999), the extent to which task differences impact the accurate measurement of interoceptive accuracy remains unknown. It is generally accepted, however, that the heartbeat discrimination task is more difficult than the heartbeat tracking task (possibly due to the requirement to integrate exteroceptive and interoceptive signals; see Pennebaker, 2012), and it is possible that age-related differences are more apparent when tasks are more demanding. Also, the present study investigated interoceptive accuracy into later stages of the lifespan than Khalsa et al., (2009). It remains a possibility that with advancing age certain individuals pay increased attention to accurate measurements of bodily sensations (e.g., monitoring blood pressure). Such individual differences in health-related behaviour may account for the increasingly varied performance observed in later life.

Whilst the present set of studies investigated interoception across the dimensions of interoceptive accuracy and awareness, these facets of interoception were not examined together. Thus, the extent to which interoceptive awareness predicts interoceptive accuracy in late adulthood remains an unanswered question. However, examining changes in metacognition for interoceptive information (the extent to which confidence in one’s interoceptive accuracy and awareness predicts interoceptive accuracy; Garfinkel et al., 2015) across the lifespan (Palmer, David, & Fleming, 2014; Vukman, 2005) may be crucial for identifying individuals at risk of illness associated with poor interoception. Whilst this study and others converge to suggest that interoceptive accuracy declines across the lifespan, if older adults are aware of this deterioration they may be more inclined to utilise external strategies for gauging interoceptive states (e.g., to ensure good hydration older adults may audit their liquid intake rather than relying on interoceptive feelings of thirst). In contrast, individuals with poor interoceptive metacognition—particularly those with inflated beliefs regarding their ability—may utilise unreliable interoceptive cues for gauging interoceptive states, placing them at a greater risk of adverse health outcomes (e.g., dehydration).

In conclusion, Study One demonstrated a negative relationship between age and interoceptive awareness. Study Two demonstrated that the decline in interoceptive accuracy across the lifespan continues into late and very late adulthood. A decline in interoceptive awareness and accuracy with increasing age highlights the importance of understanding the relationship between age-related changes in interoceptive ability and age-related changes in cognition and physical and mental health.
